# Graphene-Based Nanomaterials for Photothermal Therapy in Cancer Treatment

**DOI:** 10.3390/pharmaceutics15092286

**Published:** 2023-09-06

**Authors:** Daniela F. Báez

**Affiliations:** 1 Escuela de Medicina, Universidad de Talca, Talca 3460000, Chile; daniela.baez@utalca.cl; 2 Instituto de Investigación Interdisciplinaria, Vicerrectoría Académica, Universidad de Talca, Talca 3460000, Chile

**Keywords:** graphene oxide, reduced graphene oxide, graphene-based nanomaterials, photothermal therapy, magnetic iron oxide nanoparticles, cancer

## Abstract

Graphene-based nanomaterials (GBNMs), specifically graphene oxide (GO) and reduced graphene oxide (rGO), have shown great potential in cancer therapy owing to their physicochemical properties. As GO and rGO strongly absorb light in the near-infrared (NIR) region, they are useful in photothermal therapy (PTT) for cancer treatment. However, despite the structural similarities of GO and rGO, they exhibit different influences on anticancer treatment due to their different photothermal capacities. In this review, various characterization techniques used to compare the structural features of GO and rGO are first outlined. Then, a comprehensive summary and discussion of the applicability of GBNMs in the context of PTT for diverse cancer types are presented. This discussion includes the integration of PTT with secondary therapeutic strategies, with a particular focus on the photothermal capacity achieved through near-infrared irradiation parameters and the modifications implemented. Furthermore, a dedicated section is devoted to studies on hybrid magnetic-GBNMs. Finally, the challenges and prospects associated with the utilization of GBNM in PTT, with a primary emphasis on the potential for clinical translation, are addressed.

## 1. Introduction

**Figure 1:**
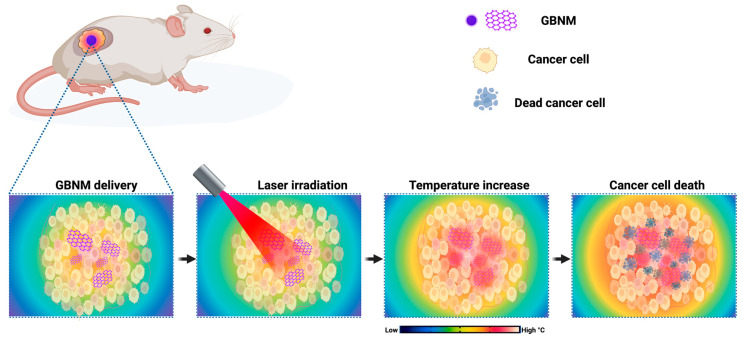
Schematic illustration of photothermal therapy for cancer treatment. This figure is original for this work. Created with BioRender.com.

Cancer is among the leading causes of death worldwide [1]. The conventional treatments for this disease include surgical resection of the tumor, hormonal therapy, radiotherapy, and chemotherapy with a combination of drugs. However, most of these strategies induce adverse reactions with varying degrees of severity in patients or low effectiveness when the disease is in advanced stages [2]. Consequently, researchers are constantly searching for noninvasive anticancer strategies. Photothermal therapy (PTT) has attracted widespread attention due to its excellent therapeutic efficacy in locally treating different types of cancer. PTT is promoted by materials termed photothermal agents (PTAs) that absorb and convert near-infrared (NIR) light into heat; this heat generates sufficient temperature to cause hyperthermia, which can induce the death of cancer cells [3,4,5]. As depicted in Figure 1, the therapeutic approach with PTT entails several sequential steps. First, a targeted PTA is administered to the tumor site. Then, NIR irradiation is locally applied to the affected area to excite the surface plasmons of the PTA, causing the absorbed energy to be converted into heat through nonradiative relaxation. Subsequently, the temperature rises, leading to hyperthermia, which selectively targets and destroys cancer cells [6,7,8,9].

A key feature of PTT is that the applied NIR irradiation must induce high photothermal conversion to produce a temperature greater than 42 °C and cause hyperthermia in the damaged zone [10]. This process becomes challenging when tumors are located deep within tissues, such as in lung, pancreatic, colorectal, and stomach tissues, or when the disease has spread throughout the body. To overcome these limitations, researchers have combined PTT with a second therapeutic strategy to improve the therapeutic effect and used nanomaterials with strong absorption of NIR light to increase the photothermal conversion [11,12,13]. Reports of these strategies have been well documented, with a focus on cancer therapy and the nature, shape, and size of the different nanomaterials applied [9,11,14,15,16,17,18,19].

Several nanomaterials exhibit remarkable NIR-absorbing capabilities from the first NIR window (NIR-I, 750–1000 nm) or second NIR window (NIR-II, 1000–1500 nm), including noble and transition metal nanoparticles [20], carbon nanotubes [21,22], and graphene-based nanomaterials (GBNMs) [23]. Among them, gold nanoparticles have been extensively studied as PTAs and have demonstrated good biocompatibility and low toxicity. However, their ability to perform photothermal conversion is influenced by the morphology, size, and specific wavelength used for the irradiation [24]. GBMNs, specifically graphene oxide (GO) and reduced graphene oxide (rGO), are 2D materials that have shown great potential in cancer therapy. Due to the outstanding physicochemical properties of GO and rGO, these materials have been applied in biosensing and in the treatment of different types of cancer [25].

The applications of GO and rGO in PTT have recently been well reviewed because these materials exhibit strong absorbance in the NIR region. These reports focused on their functionalization, their toxicity, and the type of cancer treated. Nevertheless, comparisons between the photothermal capacity of GO and rGO have been limited [11,23,25,26,27,28,29].

This review focuses on providing a comparative analysis of the photothermal capacity of GO and rGO for the therapy of different types of cancer. In the following sections, a comparative overview of GO and rGO structural features is presented through different characterization techniques. Then, the utility of GO/rGO-based systems for single photothermal and combined photothermal therapy of different types of cancer is analyzed. Emphasis was placed on the photothermal conversion obtained according to the NIR wavelength, the type of GBNMs, the modification performed, and the secondary therapy used. After that, a special section is dedicated to hybrids between GBNM and magnetic iron nanoparticles, particularly magnetite and maghemite, as these materials can potentially be manipulated by applying an external magnetic field; thus, these materials could be guided and used as photothermal agents [30,31].

Finally, challenges and prospects are discussed regarding the application of GBNMs for photothermal therapy and potential clinical use.

## 2. Graphene-Based Nanomaterials

### 2.1. Structural Features of Graphene-Based Nanomaterials

Graphene oxide and reduced graphene oxide are the two main kinds of graphene-based nanomaterials within the family of carbon nanomaterials [32,33,34]. Both GBNMs exhibit exceptional properties and high biocompatibility owing to their composition and structural features; as a result, the GBNMs are used in many biomedical applications [23]. 

**Figure 2:**
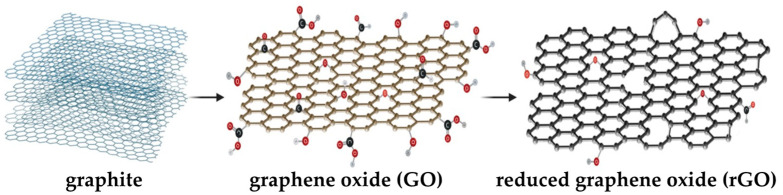
Schematic illustration of graphite, graphene oxide, and reduced graphene oxide structures. This figure is original for this work. Created with BioRender.com.

GO and rGO share similarities, as shown schematically in Figure 2. They are a 2D one-atom-thick layer that is composed of carbon atoms arranged in a honeycomb lattice with a network of delocalized π electrons. However, despite these similarities, their structures exhibit differences that affect their properties. For instance, GO is commonly synthesized through the Hummers and Offeman method [35], which consists of oxidizing graphite with a strongly acidic solution under controlled reaction conditions. Then, the oxidized graphite is subjected to a mechanical process that allows its interaction with water molecules, which intercalate and separate the sheets into random sizes to generate graphene oxide. Thus, the structure of graphene oxide changes from the original honeycomb lattice of graphite to a network of sp^2^ and sp^3^ hybridized carbon atoms and several oxygen functional groups, such as hydroxy, epoxy, and carboxy groups [36]. In contrast, rGO is obtained by reducing the oxygen functional groups in graphene oxide and restoring the sp^2^ network to a similar graphene-like structure [37]. Chemical, thermal, hydrothermal, and electrochemical methods are the most commonly used techniques to reduce GO [37]. However, depending on the chosen reduction method, different carbon, oxygen, and intrinsic defect degrees are obtained.

Compared with GO, rGO exhibits greater electrical conductivity, which is particularly beneficial for the development of electrochemical biosensors [33,38]. However, rGO is highly hydrophobic and poorly dispersed in aqueous solutions; thus, the loading of molecules is usually achieved through noncovalent interactions, i.e., π–π and electrostatic interactions. As a result, drug administration must be carefully considered due to the possibility of desorption in non-target areas. Conversely, GO is more hydrophilic than rGO, disperses better in water, and can be functionalized with various biomolecules through covalent or noncovalent interactions, making it an excellent drug carrier [39,40].

### 2.2. Structural Characterization of Graphene-Based Nanomaterials

The differences in properties between GO and rGO are mainly attributed to their composition and structure. Various characterization techniques, such as X-ray photoelectron spectroscopy (XPS), Raman spectroscopy, transmission electron microscopy (TEM), X-ray diffraction (XRD), and UV–NIR spectrophotometry, are commonly employed to determine the structure of GO and to assess the degree of reduction when rGO is formed.

#### 2.2.1. X-ray Photoelectron Spectroscopy (XPS)

**Figure 3:**
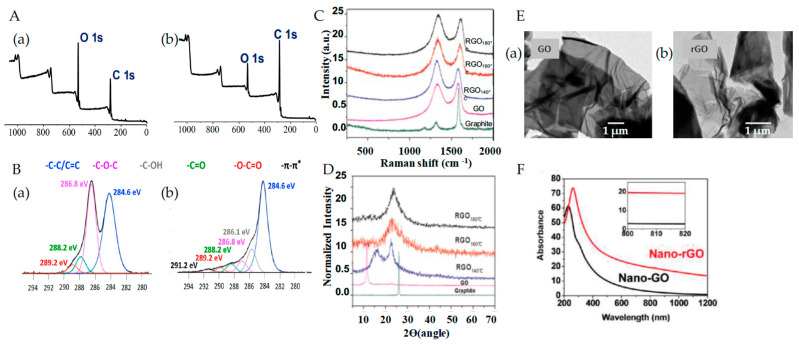
(**A**) X-ray photoelectron spectroscopy (XPS): wide scan recorded from (**a**) GO and (**b**) rGO. (**B**) XPS: C 1s spectra for (**a**) GO and (**b**) rGO. (**C**) Raman spectra of graphite (green), GO (purple), and rGO (black, red, and blue). (**D**) XRD patterns of graphite (green), GO (purple), and rGO (blue, red, and black). (**E**) TEM images of (**a**) GO and (**b**) rGO. (**F**) UV–vis absorption curves of nanoGO (black) and nanorGO (red). The inset shows a magnified view of the curves in the 800 nm region. (**A**,**B**) were adapted from Ref. [45] Copyright 2017 Nanomaterials. (**C**–**E**) were adapted from [46] Copyright 2014 Elsevier. (**F**) was adapted from [47] Copyright 2011 American Chemical Society.

The chemical composition of GO and rGO can be analyzed through X-ray photoelectron spectroscopy. Figure 3A(a,b) display the wide-scan spectra of GO and rGO, respectively. By examining the carbon and oxygen intensities, the effectiveness of a reduction method can be determined based on the C/O or O/C ratios [41,42,43,44]. A higher C/O or lower O/C ratio indicates a less prominent oxygen peak and a better reduction of oxygen functionalities to form rGO. In this regard, the atomic O/C ratio for GO was higher than that for rGO, as expected. Figure 3B(a) shows high-resolution C 1 s spectra for GO that present four distinct contributions related to the functional groups due to oxidation. The contribution located at 284.6 eV are associated with the C–C/C=C groups, whereas those observed at 286.8 eV, 288.2 eV, and 289.2 eV correspond to the C–O, C=O, and O–C=O groups, respectively. the C 1s spectra of rGO are presented in Figure 3B(b). An additional contribution was needed to accurately fit the experimental data at 286.1 eV, which is associated with the C–OH groups. Additionally, a broad π–π* satellite peak was observed at approximately 291.2 eV [45].

#### 2.2.2. Raman Spectroscopy

Raman spectroscopy is a nondestructive technique that characterizes the structure and quality of GBNMs. The most distinctive Raman modes observed for GBNMs are the D band (1324–1346 cm^−1^) and the G band (1490–1691 cm^−1^). The formation of the D-band results from the disruption of the graphitic sp^2^ backbone, and its intensity is related to the number of defect sites. Figure 3C (green curve) shows the Raman spectra obtained for pristine graphite, which displays a characteristic prominent G peak at 1585 cm^−1^ and a very weak D peak at approximately 1322 cm^−1^ caused by the graphite edges, indicating that the graphite structure is very regular. The Raman spectra for GO are exhibited in Figure 3C (pink curve) and present a D band at approximately 1332 cm^−1^ and a G band at 1579 cm^−1^. The D band is very prominent compared with that observed for graphite, indicating that the defects increase after oxidation. This is due to changes in the hybridization from sp^2^ to sp^3^ carbon atoms. A relationship between the intensities of the D and G bands (ID/IG ratio) is frequently used to quantify the degree of defects, and the ID/IG ratio of GO is higher than that of graphite. After the reduction process, the D band intensity continues to grow with respect to the G band (Figure 3C; blue, red, and black curve), and the ID/IG ratio indicates a significant degree of structural disorder from GO to rGO [46].

#### 2.2.3. X-ray Diffraction (XRD)

The X-ray diffraction patterns obtained for graphite, GO, and rGO are shown in Figure 3D. The XRD pattern of graphite powder (Figure 3D, green curve) shows a sharp main diffraction peak at 2θ = 26.50°, indicative of an interlayer distance of 0.34 nm. For GO (Figure 2G; pink curve), a complete pattern disappearance of the sharp feature peak can be observed, and a new broad peak appears near 10.27° (d-spacing 0.86 nm). The increase in the interlayer distance is due to the incorporation of oxygenated functionalities during the oxidation process and to the intercalation of water molecules between the new hydrophilic GO sheets. For rGO (Figure 3D; blue, red, and black curves), a dramatic change in 2θ angles can be observed. A broad and intense peak at 24.57° (d-spacing 0.36 nm) is obtained as a result of the reduction process, suggesting that the graphitic structure is restored. However, the width of the peak indicates that rGO shows a poorer crystalline character than graphite [46].

#### 2.2.4. Transmission Electron Microscopy (TEM)

TEM is commonly employed to analyze the morphology of GBNMs. Figure 3E(a,b) show TEM images obtained for GO and rGO, respectively. Dark and large nanosheets were observed for GO, indicating some superposed layers, while transparent, wrinkled and folded structures were observed for rGO, indicating single-layer sheets [46].

#### 2.2.5. Ultraviolet–Visible–Near-Infrared Spectrophotometry (UV–VIS–NIR)

The UV–VIS–NIR spectrophotometry technique is used to study the optical absorption properties of GO and rGO. Figure 3F shows the UV–VIS–NIR absorption spectrum obtained for GO (black curve) and rGO (red curve), in which a characteristic absorption peak at 228 nm is observed for GO. This peak is associated with π–π* transitions of aromatic C–C bonds and a small shoulder at approximately 310 nm due to the n–π* transitions of C=O bonds. In the case of rGO, a redshift in the absorption spectrum of this peak is observed, with a peak at approximately 265 nm, due to the decrease in oxygen functional groups and the increase in aromaticity upon successful reduction. In addition, the rGO band shows broad absorption throughout the visible region and a remarkable increase in the NIR absorbance compared with that of GO, indicating that rGO presents a superior capacity to absorb NIR light [47]. The presence of oxygen functional groups and defects in the structure of GO and rGO enables the absorption of NIR light, the excitation of surface plasmons, and the generation of heat by nonradiative relaxation. Taken together, these factors constitute the photothermal effect [48].

## 3. GBNMs for Photothermal Cancer Therapy

To date, several GO/rGO-based systems have been explored with the aim of producing a large photothermal effect for the treatment of cancer [27]. Factors such as the duration and intensity of external laser irradiation, as well as the modification and concentration of the GBNMs, have been considered, depending on the type of cancer treated. This section summarizes the application of GO and rGO in single or combined photothermal therapy according to the type of cancer treated.

### 3.1. Breast Cancer

**Figure 4:**
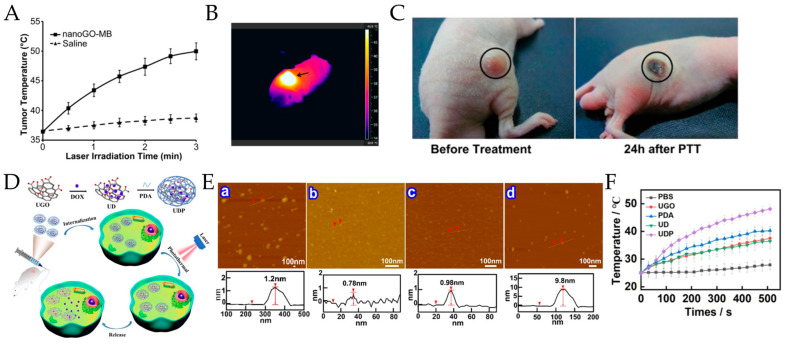
NIR-laser-induced photothermal effect of nanoGO in vivo. (**A**) The temperature change in tumor tissue after irradiation with an NIR laser (CW, 808 nm, 2 W/cm^2^). (**B**) High temperature in the tumor (indicated by black arrow) at the time of photothermal treatment, showing a major temperature difference from the surrounding body as recorded by an IR camera. (**C**) The effect of photothermal treatment on the tumor tissue shows the burning and destruction of the tissue after 24 h of laser irradiation. Reproduced from [52] (Copyright: 2013, Elsevier). (**D**) Schematic illustration of the fabrication of UDP and the combination of enhanced chemotherapy and PTT against breast cancer in vivo. (**E**) AFM height images and section analysis of (**a**) PGO, (**b**) UGO, (**c**) UD, and (**d**) UDP. (**F**) The heating curve of PBS, UGO, PDA, UD, and UDP under NIR laser irradiation (808 nm, 1.5 W/cm^2^, 500 s). Adapted from [57] Copyright 2023, Elsevier.

Breast cancer is an aggressive disease that frequently results in metastasis, which spreads the disease to surrounding tissues [49]. To improve the treatment efficiency for this cancer, researchers have combined chemotherapy and photothermal therapy into one system. For instance, Zhang et al. combined chemotherapy and PTT by modifying nanoGO with doxorubicin and PEG to create NGO-PEG-DOX. A significant increase in the temperature from approximately 26 °C to 50 °C was observed when EMT6 tumor samples were treated with NGO-PEG-DOX for 3 min under NIR irradiation (808 nm laser + 2 W/cm^2^). The inhibition rate was superior when the modified GO was used at 10 mg/mL DOX and NIR irradiation in comparison to that of free DOX and NGO-PEG. The authors attributed the enhanced cell-killing effect to the hastened DOX release from NGO-PEG at elevated temperatures and the increasing heat sensitivity of cells. The in vivo results demonstrated that when mice were treated only with DOX, the volume of their tumors rapidly grew. For mice treated with NGO-PEG without DOX and 2 W/cm^2^ NIR laser for 5 min, the tumor volume was reduced in the first week, but the tumor began to grow again. On the other hand, four out of five mice in the NGO-PEG-DOX group achieved total tumor ablation 1 day after NIR irradiation, leaving black scars on the original tumor sites. Moreover, the tumors did not regrow within the next 40 days. These findings suggest that combining chemotherapy with locally external NIR photothermal therapy could be a promising treatment option for patients with breast cancer [50]. Similar results were described by Zhu et al., who developed a thermosensitive hydrogel based on chitosan and graphene oxide loaded with docetaxel (DTX–GO/CS). When the DTX-GO/CS gel was used in combination with NIR laser irradiation (at 808 nm and 2.5 W), this was found to exhibit a higher inhibition rate in MCF-7 cells than that achieved without NIR irradiation. In addition, the effectiveness of DTX-GO/CS in reducing tumor growth was observed in S180 tumor-bearing mice after 12 days of treatment. The results revealed that compared with other control methods, the application of the nanosystem in combination with NIR irradiation resulted in a significant reduction in tumor volume and weight, suggesting that the nanosystem can effectively inhibit tumor growth [51]. The synergistic combination of PTT and photodynamic therapy (PDT) as a second therapeutic strategy has also been studied, showing that the tumor temperature reached almost 50 °C after 3 min of laser exposure when using a nanographene oxide sheet modified with the Pluronic block copolymer and complexed with methylene blue (Figure 4A,B). Tumor tissues were completely burned without mass detected on Day 3 (Figure 4C); however, a small tumor mass was observed on Day 6, and tumors started to regrow after Day 9, indicating that PTT was not enough to eradicate carcinogenic tissue. Therefore, PTT was combined with PDT. After 15 days of treatment, no tumor tissue was detected in any treated mice, indicating complete tumor regression. These results corroborate that combining PTT with a second therapy enhances in vivo cancer therapeutic efficiency [52].

The chemistry, morphology, and size of GBNMs can affect the photothermal conversion and, consequently, the results of photothermal therapy for cancer [53,54]. In this sense, Yang et al. compared the size and photothermal capacity of GO, chemically reduced GO, and their nanosized versions functionalized with C_18_PMH-PEG. The size of the nanoGO-PEG sheets was much smaller than that of rGO-PEG and similar to that of the nanorGO-PEG sheets, with equivalent diameters of 23, 65, and 27 nm. However, the NIR absorption at 808 nm of nanorGO-PEG and rGO-PEG was 3-4-fold higher than that of nanoGO-PEG. On the other hand, the in vivo results obtained using a lower power density of 0.15 W/cm^2^ and 5 min of laser irradiation showed that the surface temperature of mouse tumors treated with nrGO-PEG reached ∼48 °C, while that of tumors treated with nGO-PEG only increased by 41 °C. Moreover, the latter conditions resulted in rapid tumor growth, indicating that the unreduced version in these conditions was not effective for the photothermal ablation of tumors [55]. Li et al. carried out a similar study but compared the size and oxidation degree of GO after successive modifications for integrated chemotherapy and PPT against 4T tumors (Figure 4D). Ultrafine GO nanosheets (UGO) presented a width of 30 nm with a thickness of 0.78 nm (Figure 4F(b)), which did not significantly change after DOX loading (Figure 4F(c); UD). However, a larger average lateral size (~50 nm) and thickness (9.8 nm) was observed when polydopamine coated the UD system (Figure 4F(d); UDP). The photothermal conversion profile shown in Figure 4G presented a similar rise in temperature for UGO and UD, which reached approximately 37 °C after NIR irradiation (808 nm, 1.5 W/cm^2^, 300 s). The temperature increased dramatically when using UDP and reached approximately 50 °C, demonstrating that the enhanced photothermal effect was due to polydopamine rather than GO, even at ultrasmall sizes. Similar results were described by Hashemi et al., who showed that rGO was 3.2-fold stronger in absorbing light at 808 nm than GO. In the same study, another factor that influences photothermal conversion was investigated. The photothermal performance showed that at different power intensities and concentrations for GO and rGO, the temperature increased as the power intensity used for both GBNMs increased. However, after 5 min of irradiation with a power density of 1.7 W/cm^2^ in a 400 μg/mL GO suspension, the temperature increased to 46.2 °C; in an rGO suspension 4 times less concentrated, the temperature increased to 42.7 °C [56]. Therefore, rGO is a more effective transducer for photothermal therapy.

Several hybrid combinations of GO with other NIR light-absorbing nanomaterials have been tested for their ability to increase the photothermal response. Gold nanoparticles, which exhibit optical properties, have been utilized in cancer therapy previously [20,58]. In this sense, Wang et al. integrated gold sphere nanoparticles with GO modified with an MUC1 aptamer and loaded them with DOX (GO-AuNP-Apt-DOX) for targeted chemotherapy and PTT. The photothermal conversion efficiency was determined by irradiating different concentrations (2.7, 5.3, and 8 mg/L) of the GO-AuNP solution at different times. The results showed a dose dependency and the highest temperature was 48 °C. After exposure to NIR light, the release of DOX exceeded 80% within 2 h. In contrast, without irradiation, it only reached 40% after 7 h at 25 °C. The results obtained from the cell viability analysis on the MCF7 cell line revealed that GO-Au-Apt caused more cell death than GO-AuNPs when exposed to NIR light. However, NIR irradiation did not result in increased cell death. The MCF7 cells exhibited a viability of 90% after 5 min of irradiation, but this value decreased rapidly to 80% as the irradiation time increased to 15 min [59]. Similar results have been obtained using polymers such as poly(allylamine hydrochloride) [60] and dopamine [61]. In the last study, Lima Sousa et al. evaluated the phototherapeutic capability of GO reduced with dopamine (P-DOPA-rGO) using 3D heterotypic spheroids. When spheroids were treated with P-DOPA-rGO and NIR irradiation (808 nm, 1.7 W/cm^2^, and 5 min), the temperature increased by approximately 36 °C. However, the viability decreased only to 30%, which was lower than the results obtained with monolayers of cancer cells (3%). The authors attributed this result to the spheroids’ resistance to penetration or temperature-mediated death. These results suggest that a higher laser wavelength or concentration of the system may be necessary to reach the innermost part of this kind of 3D model tumor.

### 3.2. Lung Cancer

**Figure 5:**
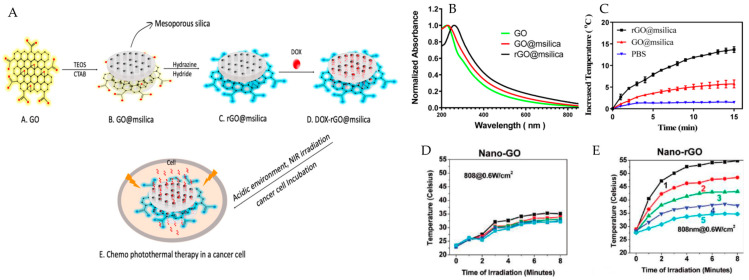
(**A**) Schematic illustration of a DOX-loaded rGO@msilica nanocarrier as a multifunctional drug delivery system for the synergetic chemophotothermal therapy of cancer. (**B**) Absorption spectra of GO, GO@msilica, and rGO@msilica nanocarriers. (**C**) Temperature-increasing curves of PBS, GO@msilica nanocarrier (0.5 mg/mL), and rGO@msilica nanocarrier (0.5 mg/mL) solutions exposed to an NIR laser (808 nm, 0.3 W/cm^2^) for 15 min. (**D**) Photothermal heating curves of nanoGO and nanorGO solutions. The black curve is 100 μL of solution with a 20 mg/L concentration of nanorGO or nanoGO; the red curve is 10 mg/L; the green curve is 5 mg/L; the dark blue curve is 2.5 mg/L; and the light blue curve is water. Panels A to C were adapted from [64] (Copyright 2020, American Chemical Society). (**D**,**E**) were adapted from [47] Copyright 2011, American Chemical Society.

The photothermal agent concentration and the intensity of the laser power are key factors that affect the efficacy of photothermal therapy. In this sense, Du et al. investigated the temperature increase for an AS1411 aptamer and berberine-derivative graphene oxide-based system at different concentrations during NIR irradiation (808 nm and 2.5 W/cm^2^). The temperature at 128 μg/mL and 10 min irradiation reached 51.2 °C. In contrast, at 32 μg/mL, only 38.6 °C was achieved, confirming the concentration dependency. Then, the therapeutic efficacy of the combined chemo- and photothermal therapy was evaluated by treating A549 and L929 cell lines at different GO and AS1411-GO concentrations. For untreated A549 and L929 cells, irradiation for 3 min did not decrease cell viability, but when GO and AS1411-GO reached the highest concentration, the cell survival rates of A549 cells and L929 cells remained at approximately 98%, indicating that GO and AS1411-GO exhibited almost no cytotoxicity. The combined chemophotothermal therapy achieved an improved therapeutic effect with AS1411-GO/B3, as the survival rate of A549 cells reduced from 51% (without NIR irradiation) to 28% (with NIR irradiation) [62]. Wang et al. also assessed how much heat could be produced under exposure to NIR light under similar conditions to those in a previous report (808 nm, 2 W/cm^2^, 5 min) but using different concentrations of rGO. The findings showed that as the concentration of rGO increased, the temperature also increased; at a concentration of 1 mg/mL and 5 min of irradiation, the temperature reached 50 °C, surpassing the photoablation temperature. In this case, the tumors from the A549 cell line treated with rGO decreased the cell viability sharply to 35% from 65% as the concentration increased [63]. In a different study, rGO was used in combination with mesoporous silica to form a sandwich structure modified with DOX (Figure 5A) in human lung cancer (A549) and human colorectal carcinoma (SW620) cell lines, and the results showed high viabilities of 84.7% with SW620 and 95.6% with A549, even at a concentration of 2.5 mg/mL. The synergistic chemophotothermal therapeutic effect was better when reduced GO (rGO@msilica) was used than when GO@msilica nanocarrier was used at a lower power intensity (0.3 W/cm^2^ for 15 min using 808 nm laser light), which showed increased NIR absorption (Figure 5B) and a remarkable temperature increase (Figure 5C) of 14 °C compared with 4 °C, respectively [64].

### 3.3. Glioma

Deep cancerous tumors, such as glioma, need to be irradiated with high-intensity light to produce hyperthermia, especially when the first NIR window is used as the source of irradiation light. In a study by Dong et al., a higher power intensity of 2.5 W/cm^2^ with a laser wavelength of 808 nm was used for combined chemophotothermal therapy. The viability of the C6 and BMVE cell lines remained above 95%, even at 100 μg/mL GO, indicating that GO is a safe nanocarrier for drug delivery. The in vivo results showed that using PEG- and DOX-based systems loaded with transferrin increased the head temperature of glioma-bearing rats by 4 °C (from 34.15 to 38.10 °C) in the focal region; this resulted from the heat conversion generated by the NIR-absorbing TPGD. By the end of the study, the median survival time of the TPGD+NIR group was significantly longer than that of the other groups (36 vs. 20 days) [5]. On the other hand, Robinson et al. used a laser of 808 nm at a very low power intensity (0.6 W/cm^2^) to irradiate different solutions of GO and its reduced version. As shown in Figure 5D, the temperature for GO remained below 36 °C after 8 min of irradiation for all the concentrations analyzed. However, when GO was reduced chemically to produce rGO (Figure 5E), the temperatures exceeded the photoablation limit of 50 °C after 5 min of irradiation under the same experimental conditions [47]. This behavior has also been observed by other authors [33], such as Cheon et al., who developed DOX-loaded BSA-functionalized rGO (DOX-BSA-rGO) nanosheets for the chemophotothermal therapy of brain tumor cells. During testing, it was found that the temperature of GO did not change significantly when exposed to a 5.5 W/cm^2^ NIR laser with a wavelength of 808 nm for 300 seconds. However, when BSA-rGO was used, the temperature exceeded 60 °C [31].

It is evident that reducing GO leads to an enhanced photothermal capacity, regardless of the method applied. Various techniques have been devised for reducing GO [33,65], all of which leave residual oxygen groups and defects that remain in the structure [45]. However, the relationship between the method used to synthesize rGO and the photothermal capacity obtained has not been well researched. A chemical reduction, for instance, was performed on a GO nanomesh to produce the unreduced (GONM) and its reduced version (rGONM), suggesting that the reduction can provide more low-energy vibrational modes and higher NIR absorption; as a result, ultralow concentrations and lower laser power intensities could be used for PTT. As expected, the rGONM-PEG exhibited excellent photothermal heating under 808 nm of irradiation at low-power irradiation (0.1 W/cm^2^), reaching 50 °C after only 6 min of continuous irradiation. The in vivo results showed that the U87MG tumor was completely eliminated when 200 μL at 1 mg/mL per mouse (corresponding to a dose of ≈10 mg/kg) of rGONM-PEG-Cy7-RGD was injected and irradiated, without significant tumor regrowth and survival over 100 days [66].

### 3.4. Prostate Cancer

Prostate cancer is a silent and frequent cancer that is the fifth leading cause of cancer death among men [1]. To improve the detection and treatment of this disease, Zhang et al. designed and developed the GO/Bi_2_Se_3_/polyvinylpyrrolidone (GO/Bi_2_Se_3_/PVP) system by using the solvothermal method. Then, X-ray computed tomography and photoacoustic (CT/PA) imaging combined with PTT was used to study the GO/Bi_2_Se_3_/PVP system. This study demonstrated that using an 808 nm laser at 0.4 W/cm^2^, the temperature of a solution containing GO/Bi_2_Se_3_/PVP increased depending on the concentration and duration of laser irradiation exposure. After 5 min of irradiation, the temperature increased by up to 33 °C in a solution containing 150 µg/mL GO/Bi_2_Se3/PVP. The researchers believe that this enhanced photothermal effect was due to the in situ reduction of GO during the preparation [67]. A system similar to the previous one, in which a hybrid approach is used for multimodal imaging and photothermal therapy, was created using the solvothermal method. However, in this case, BaGdF_5_ was used as the contrast agent. The temperature increase was 33 °C, achieved within 10 min at a concentration of 200 µg/mL [68].

A study conducted by Thapa et al. demonstrated that pegylated graphene oxide and a polar lipid liquid crystalline nanoparticle named monoolein (LCN) could potentially serve as a nanocarrier for docetaxel (PEG-GO/LCN/DTX) in chemophotothermal therapy. The system was subjected to high-intensity irradiation of 3 W/cm^2^ for 3 min, which raised the temperature to 50 °C, resulting in increased cytotoxicity of the formulation. In vitro cell studies were carried out using DU145 prostate cancer cells, which are known for their DTX resistance and high metastatic potential. The results showed that the formulation exhibited high cellular uptake and inhibitory effects on the motility of DU145 cells [3]. SreeHarsha and colleagues also achieved similar outcomes using chitosan as a stabilizing matrix. They developed a nanocarrier system (HNP) that loaded DOX onto reduced graphene oxide. When PC-3 cell lines were treated with rGOD-HNP and irradiated, the toxicity was 2.53 times higher than that of rGOD-HNP without laser irradiation [69]. 

**Figure 6:**
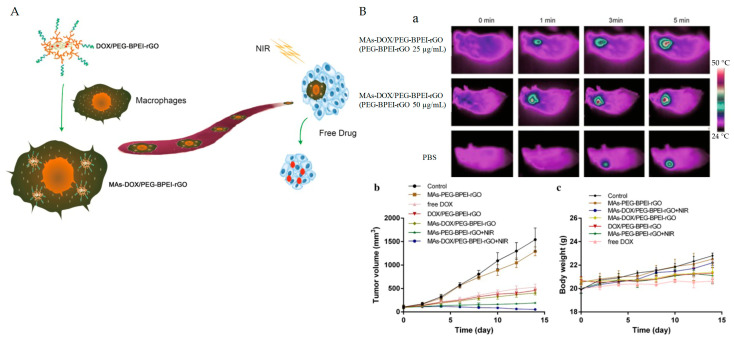
(**A**) Schematic illustration of macrophage loading with DOX/PEG-BPEI-rGO to target the tumor site and initiate drug release after NIR irradiation. (**B**) Thermographic images of tumor-bearing mice treated with PBS or MAs-DOX/PEG-BPEI-rGO 4 h after injection following NIR irradiation for 5 min (1 W/cm^2^). (**b**) Relative tumor volume in different treatment groups (n = 5). (**c**) Changes in body weight in response to different treatments (n = 5). Adapted from [70] Copyright 2019, Lei Qiang et al., Springer Nature.

A more recent study examined a nanocarrier that uses macrophages as a biomimetic drug delivery system. This approach combined the natural function of macrophages with DOX, rGO, and NIR irradiation to destroy the macrophages and excrete DOX into the surrounding environment as a free drug. Figure 6A illustrates this process. Figure 6B(a) shows the photothermal conversion efficiency of MAs-DOX/PEG-BPEI-rGO, which produced a temperature of 55.8 °C 5 min after NIR irradiation is applied using an 808 nm laser (1 W/cm^2^) at 50 μg/mL. The study investigated the antitumor efficacy of this approach in tumor-bearing mice. The tumor temperature increased to 46.3 °C after treatment with MAs-DOX/PEG-BPEI-rGO (50 μg/mL PEG-BPEI-rGO) and NIR laser irradiation. Due to the good photothermal conversion efficiency of the rGO-based system, the growth of tumors (Figure 6B(b,c)) was significantly suppressed. In contrast, tumors in the group without NIR irradiation showed a growth rate similar to that in the control group. Furthermore, compared with the group treated with the same system without DOX but with irradiation, the group treated with MAs-DOX/PEG-BPEI-rGO and irradiation showed a better tumor inhibition effect [70].

Other hybrids have been formed with GO and rGO to improve photothermal conversion, including gold nanorods [71], graphene quantum dots [72], artesunate [73], and alginate [74], among others [75,76]. Table 1 summarizes the studies in which hybrids between GO/rGO and different materials with photothermal properties were used to improve the photothermal efficiency in the photothermal treatment of cancer. It is worth mentioning that many of these studies use laser light from the NIR-I window (mostly 808 nm) to induce hyperthermia. Although this wavelength is attractive because biological systems lack chromophores that absorb in this region, it is not very effective in treating tumors that are deep or sizable because its penetration depth in body tissues is limited; for that reason, higher power intensities are required. In this sense, the NIR-II window is a better option to use as a light source for irradiation because it can penetrate deeper into tissues due to less scattering with longer wavelengths. Additionally, higher power intensities can be used [48,77,78]. Therefore, when designing new strategies to treat cancer photothermally using GBNMs, researchers should consider the NIR-II region for irradiation.

### 3.5. Liver Cancer

By incorporating transition metals or polymer biomolecules, GBNMs can be combined to create hybrid functional materials that can greatly improve their photothermal properties [91]. For instance, Liu et al. utilized polydopamine to create a hybrid material consisting of reduced graphene oxide and mesoporous silica nanomaterial (rGO/MSNs/PDA), which was then loaded with DOX. The presence of MSNs in this system increased its drug-loading capacity, while both rGO and PDA enhanced its photothermal capacity. The results showed that rGO/MSN/PDA produced a 62.2% higher temperature postirradiation than that by GO/MSNs with the same mass concentration. The comparison tests conducted on MHCC-97L and MHCC-97H hepatocellular carcinoma cells showed that rGO/MSNs/PDA were more biocompatible than GO/MSNs in regard to in vitro cytotoxicity. On the other hand, the cell activity of MHCC97L and MHCC97H in the irradiated groups was 42.4% and 44.2% lower than that in the nonradiated groups, indicating that photothermal therapy significantly improves the antitumor effect compared with chemotherapy alone [92]. Huang et al. conducted a study wherein they synthesized a nanohybrid by combining graphene oxide and indocyanine green modified with lactobionic acid, creating a double photothermal agent. This nanohybrid was employed in a mediated synergetic chemophotothermal therapy approach. The research revealed that as the irradiation time was prolonged, the temperature exhibited a corresponding increase. Remarkably, a photothermal conversion capability of 16.6 °C was achieved within just 5 min. In vivo assays demonstrated that this system led to an increase in temperature of 52.9 °C, which induced local hyperthermia, specifically within cancer cells. Simultaneously, the hyperthermic environment and the tumor’s acidic microenvironment facilitated the release of drugs. The strategy proved to be highly effective in eradicating cancer cells and curbing tumor growth [93].

Transition metals have also been used in combination with reduced graphene oxide, generating increased photothermal conversion for this type of cancer [94]. In this work, a 980 nm laser at 1.0 W was used to irradiate a Cu2−xSe@rGO system coated with PAH, folic acid (FA), and DOX. A temperature increase from ∼25 to 55 °C was obtained after 10 min of irradiation. On the other hand, no obvious cytotoxicity was observed when the concentrations of Cu2−xSe@rGO were lower than 75 μg/mL. The cell viability showed a significant decrease after NIR irradiation, and the inhibition ratio against HEp-2 cells increased with Cu2−xSe@rGO-FA concentration. 

### 3.6. Pancreatic Cancer

Eco-friendly methods for reducing GO have been investigated as an alternative to conventional methods that involve toxic chemicals. One of them is the reduction using an extract of *Salvia spinosa*. Yang et al. used the bioactive components of the extract of *Salvia spinosa* to produce rGO and evaluated its photothermal efficacy at different concentrations and power densities. The results obtained in the study showed that unmodified rGO achieved a maximum temperature increase after 4 min of laser irradiation at 808 nm with a power density of 1.7 W/cm^2^. The treatment of the Panc02-H7 PC cell line with laser light alone did not result in significant cell death. However, after laser irradiation, the rGO-treated groups showed higher levels of cell death in comparison to GO, even after increasing the GO concentration four-fold [95]. Wu and their team utilized a traditional method of reducing GO using hydrazine. The produced rGO was modified with C18-PMH-mPEG5000, and the photothermal capacity was examined by exposing it to NIR irradiation from the second window (980 nm laser) at varying intensities. In particular, when the system was used at a concentration of 50 µg/mL and power intensity of 1.5 W/cm^2^, it was able to reach a temperature of almost 80 °C. The results from the in vivo photothermal study, utilizing a lower laser power intensity of 0.5 W/cm^2^ along with 2 mg/kg of modified rGO, revealed that tumors located in the center and bottom regions exhibited a temperature of approximately 68 °C. The findings of this work demonstrate that, with a higher laser wavelength, it is possible to attain deeper therapeutic temperatures [96].

Among the studies that examined GO for pancreatic cancer, Ying et al. conducted a study that employed a combination of photothermal therapy and gene therapy through modification with small interfering RNA (siRNA). The outcomes of their in vivo antitumor investigation demonstrated that utilizing GO-based nanoformulations in tandem with near-infrared light led to a remarkable reduction in tumor volume growth, achieving up to an 80% decrease. Notably, the synergistic effects of GO-siRNA nanoformulations and NIR-light treatment resulted in the complete remission of tumors in one-third of the experimental mice. Regarding toxicity, in vivo assessments indicated that GO exhibited minimal harmful effects. However, it is crucial to highlight that the intravenous administration of folic acid-GO nanoparticles resulted in rapid mortality among mice, while intraperitoneal injection showed no side effects or associated fatalities [95].

### 3.7. Ovarian Cancer

In an emerging treatment to improve the therapeutic efficacy against ovarian cancer, a 1,061 nm diode laser (spot size: 1 cm) with different power densities was used. The temperature of the hybrid between gold nanoparticles coated with reduced graphene oxide (rGO-AuNPs) was approximately 61 °C, which was higher than that of AuNPs (15 °C) and rGO (33 °C). The rGO-AuNPs enabled high tumor accumulation post-intravenous-injection into SKOV-3 tumor-bearing mice, resulting in an intensive photoacoustic (PA) signal. More importantly, the PA signal was observed only in the tumor tissue, and no background signal was present in the skin, indicating the higher contrast and resolution of the NIR-II PA imaging of the rGO-AuNP compared with conventional NIR-I PA imaging. Finally, compared to the continued tumor growth in other control groups, the tumors in mice treated with the rGO-AuNP postcombinatorial treatment with PA and PTT were effectively eliminated without recurrence [97].

### 3.8. Tongue Squamous Cancer

**Figure 7:**
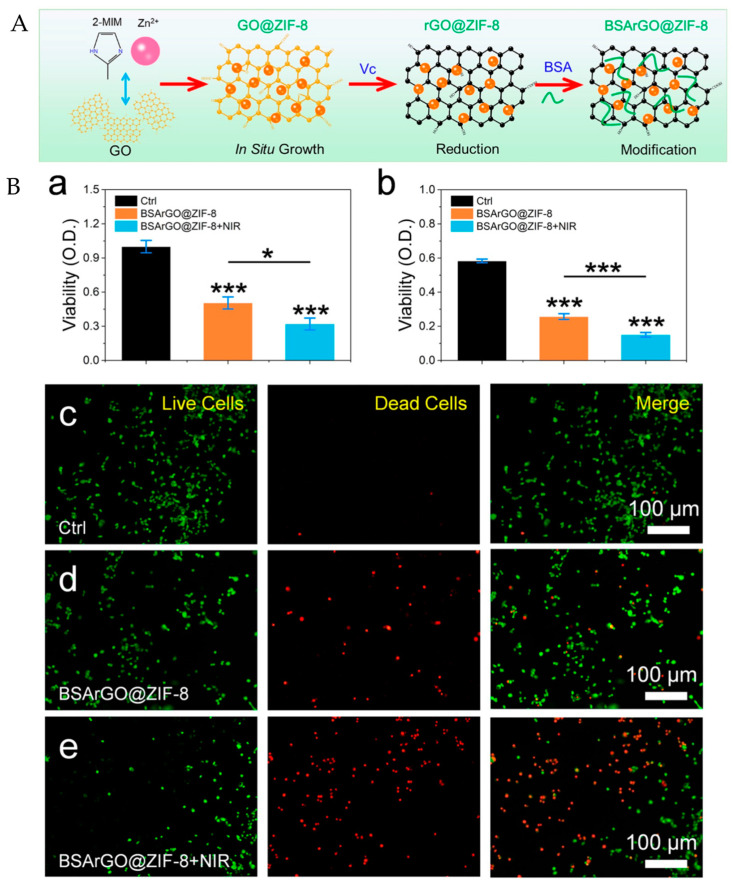
(**A**) Schematic preparation of BSArGO@ZIF-8 NSs. (**B**) Synergistic effect of PTT and IIT. (**a**,**b**) Viability of SCC25 cells and Cal27 cells after BSArGO@ZIF-8 NSs treatment with or without NIR irradiation, respectively (* *p* < 0.05, *** *p* < 0.001). (**c**–**e**) Live/dead staining of Cal27 cells treated with BSArGO@ZIF-8 NSs with or without NIR irradiation. Adapted from [99] Copyright 2022, American Chemical Society.

Hao et al. utilized tea polyphenol to reduce and functionalize GO (TPG). Then, a new and versatile anti-PDL1-conjugated TPG (TPDL1) loaded with DOX was developed. Cell-death-ligand 1 (PDL1) is a specifically expressed cell membrane antigen that shows high expression in cancer cells and low expression in normal cells. The reduced version of GO presented high photothermal conversion efficiency, reaching 60 °C after 9 min of irradiation. Additionally, after three repeated cycles of irradiation for 3 min followed by cooling for 3 min, the system showed reversible photothermal stability. The release of DOX from TPDL1 was pH-dependent, and at pH 5.0 and 48 h, it reached 22.60% release. However, the photothermal effect increased the DOX release percentage from 13.26% (−NIR) to 45.30% (+NIR) at pH 5.0 for only 6 h [98]. A similar photothermal response was obtained in a system based on rGO modified with bovine serum albumin used as a carrier nanoplatform for zeolitic imidazolate framework-8 (BSArGO@ZIF-8) for combinatorial ion interference (IIT) and PTT (Figure 7A(a)). The system serves as an efficient Zn^2+^ source that can disrupt intracellular homeostasis, causing an increase in the level of reactive oxygen species (ROS), mitochondrial damage, and cell apoptosis. The authors state that GO should be reduced to increase the photothermal effect. In this study, the cell viability of SCC25 cells after BSArGO@ZIF-8 NSs and NIR irradiation decreased to ∼15% (Figure 7B(a)). Similarly, the viability of Cal27 cells was approximately ∼20% after irradiation (Figure 7B(b)). However, NIR irradiation alone at 808 nm (1 W/cm^2^) for 10 min had negligible effects on Cal27 cells. Furthermore, live/dead staining of Cal27 cells was performed. Figure 7B(d)) shows that many dead cells were imaged after BSArGO@ZIF-8 NSs treatment compared with the control group (Figure 7B(c)). After NIR irradiation (Figure 7B(e)), more dead cells emerged, and few live cells could be observed [99]. 

### 3.9. Hybrid Magnetic Graphene-Based Nanomaterials

Magnetic nanoparticles (MNPs) [30,100], including magnetite (Fe_3_O_4_) and maghemite (Fe_2_O_3_), can be magnetically controlled by an external magnetic field, making them valuable for medical applications [101,102,103]. In this sense, Dash et al. developed a hybrid between rGO and magnetic nanoparticles (mrGO) loaded with DOX (mgGOG) that exhibited excellent magnetic and photothermal properties for targeted drug delivery and PTT. The SQUID analysis confirmed the superparamagnetic properties of all the samples, with remanent magnetizations of 0.14, 0.04, and 0.03 emu/g for CMNPs (citrate-coated magnetic nanoparticles), mrGO, and mrGOG, respectively. The photothermal results obtained using an 808 nm NIR laser (1.5 W/cm^2^) showed that mrGO possesses a higher photothermal response than rGO, reaching 60 °C at 150 s. This temperature supports previous findings that iron oxide nanoparticles have a photothermal response after NIR irradiation [104]. Guided drug delivery was confirmed by incubating mrGOG with U87 cells under the guidance of a magnet glued to the bottom of the well surface. The results obtained from the live/dead cell viability staining assay revealed that dead cells, marked by red fluorescence, accumulated in the magnetically targeted zone at which mrGOG/DOX was attracted and concentrated, causing cell death. However, the density of dead cells was lower than expected due to their detachment from the well surface after cell death. On the other hand, live cells, identified by green fluorescence, were mainly located outside the targeted zone and lacked magnetic guidance. This suggests that an external magnetic field could guide mrGOG/DOX to the tumor site, increasing the drug concentration and offering the potential for dual-targeted anticancer therapy in vivo [105]. Ardakani et al. used two different concentrations of reduced graphene oxide to increase the therapeutic efficiency of Fe_3_O_4_@Au nanoparticles in the in vitro photothermal radiotherapy of KB oral squamous carcinoma. The photothermal conversion efficiency results showed that Fe_3_O_4_@Au/rGO nanostructures (NSs) under 808 nm laser irradiation and 1.8 W/cm^2^ had a high photothermal conversion efficiency (61%) and were very suitable for photothermal applications. Viability assays showed that using NSs+PPT is approximately 13 times more effective than using the control group (without NIR) [106]. Another hybrid between magnetite and rGO without other modification was prepared to investigate the photothermal capacity at two concentrations (50 and 100 µg/mL), irradiating them with an 804 nm optical laser at 1 W/cm^2^ for 5 min. A similar increase in the temperature was observed for both concentrations. The authors suggest that this could be associated with the insoluble nature of the hybrid or the incomplete reduction of GO that also influences the lower temperature reached. In vitro results showed that photothermal therapy with the hybrid reduced cell viability to 32.6% and 23.7% at 50 and 100 µg/mL, respectively, while untreated cells were not noticeably affected; even under laser exposure, viability was maintained at over 83% [107]. Soysal et al. prepared the same hybrid between magnetite and rGO but modified it with polyaniline (S-rGO-Fe3O4-PANI), with excellent photothermal performance. The maximum temperature achieved after 10 min of long irradiation at 808 nm and 3.0 W/cm^2^ was ∼60 °C. At a laser power density of 2.0 W/cm^2^ and a concentration of 100 μg mL^−1^, the temperature of the nanocomposite increased by 56.7 °C in comparison to deionized water, and an 86.3% photothermal conversion efficiency was obtained [108]. 

Du et al. used rGO to anchor iron oxide, polyethyleneimine (PEI), and indocyanine green (RGI_1.8k_-ICG) as a drug model. The hybrid did not exhibit significant cytotoxicity and showed good biocompatibility. The level of cell destruction varied with different laser densities; for example, at laser densities lower than 0.3 W/cm^2^, no cell destruction effects were observed for the PBS, ICG, RGI_1.8k_, and RGI_1.8k_-ICG groups. However, it decreased rapidly for the RGI_1.8k_-ICG group when the laser density reached 0.5 W/cm^2^ and for the RGI_1.8k_ group when it reached 0.7 W/cm^2^. The in vivo results obtained using a laser density of 0.3 W/cm^2^ produced a temperature at the tumor site of approximately 55 °C, which was sufficient to ablate the cancer cells efficiently and cause the near disappearance of the tumor. However, without laser irradiation, the tumor grew rapidly and became almost as large as that of the negative control group after 15 days [109].

The photothermal effect of GO functionalized with Fe_3_O_4_ (MG-NH2-PEG) and DOX was also evaluated in the treatment of breast cancer. A rapid increase in the temperature pursuant to a concentration-dependent behavior was obtained when an 808 nm NIR laser was used with a power density of 1 W/cm^2^. The MCF-7 cell line was incubated with MG-NH2-PEG for 2 h and irradiated for 5 min with varying power densities. Then, in vitro cytotoxicity tests were performed, showing that the cells were killed after laser irradiation. The untreated cells maintained their viability despite the higher laser irradiation (up to 2 W/cm^2^). Furthermore, the cells treated with 20 min of NIR laser irradiation closer to the magnetic field were significantly destroyed, while those located at a further distance remained largely unaffected [110].

**Figure 8:**
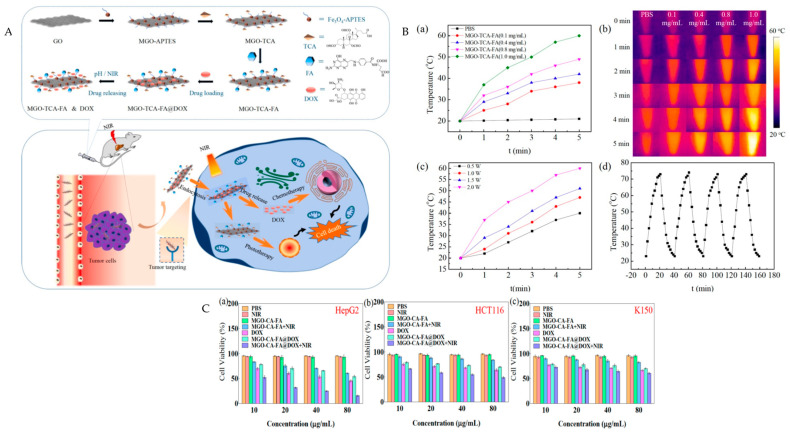
(**A**) Schematic diagram of MGO-TCA-FA@DOX and its photochemotherapy effect on cancer cells. (**B**) Temperature-change curves (**a**) and corresponding infrared thermal images (**b**) of MGO-TCA-FA solutions at different concentrations obtained by using the thermal imaging system; (**c**) temperature-rise curve of MGO-TCA-FA irradiated with different laser irradiation intensities (808 nm); (**d**) temperature-variation curve of MGO-TCA-FA with 20 min laser on/off cycles. (**C**) Cell survival rate of HepG2 (**a**), HCT-116 (**b**), and K150 (**c**) cells treated with PBS, PBS + NIR, MGO-TCA-FA, MGO-TCA-FA + NIR, DOX, MGO-TCA-FA@DOX, and MGO-TCA-FA@DOX + NIR. The concentrations of MGO-TCA-FA and MGO-TCA-FA@DOX were 10, 20, 40, and 80 μg mL^−1^, respectively. Adapted from [111] Copyright 2021, Elsevier Inc.

Gong et al. prepared a novel multifunctional hybrid material, which is depicted in Figure 8A. The MGO-TCA-FA hybrid synthesis process involves modifying magnetic graphene oxide (MGO) with triformyl cholic acid (TCA) and folic acid (FA) simultaneously. The hydrophobic anticancer drug DOX was then successfully loaded onto the modified MGO to create MGO-TCA-FA@DOX nanocomposites. This hybrid was effective in providing chemotherapy and photothermal therapy for liver cancer. Figure 8B(a) shows that when exposed to 808 nm and 2 W/cm² for 5 min, the solution temperature increased from 21 °C to 60 °C with the concentration from 0 mg/mL to 1.0 mg/mL. Figure 8B(b) shows the respective thermal images obtained. In addition, MGO-TCA-FA also exhibited laser power intensity dependency (Figure 8B(c)), and, more importantly, it had good photothermal stability after four 20 min laser on/off cycles (Figure 8B(d)) [111].

## 4. Challenges and Perspectives

Methods to utilize graphene nanomaterials in biomedical applications have advanced significantly in recent years. GO and rGO have been identified as potential photothermal agents in nanomedicine due to their physicochemical properties and high biocompatibility. However, there are still several issues that need to be addressed before photothermal therapy based on these nanomaterials is implemented in clinical trials, especially when tumors are located deep within tissues or when metastasis is spread throughout the body.

First, many studies reviewed here have focused on using laser light from the NIR-I window (mostly 808 nm) to induce hyperthermia. Although this wavelength is attractive because biological systems lack chromophores that absorb in this region, thus reducing scattering and autofluorescence, it is not very effective in treating tumors that are deep or sizable because its penetration depth in body tissues is limited. This issue is particularly important in clinical settings because if the method is combined with GO as PTA, photothermal therapy will be inefficient. For that reason, the NIR-II window is a better option because it can penetrate deeper into tissues due to less scattering with longer wavelengths. Additionally, higher power intensities can be used since longer wavelengths have less energy per photon [6,10,14,48,77]. Therefore, when designing new strategies to treat cancer photothermally using GBNMs, researchers should consider the NIR-II region for irradiation.

Another critical issue to consider when designing clinical trials is the potential harm caused by GBNMs, modified GBNMs, and NIR irradiation to human health. The toxicity of GBNMs can be influenced by a range of factors, including their size, chemical composition, surface charge, and aggregation state [13]. Although GO and rGO have exhibited strong biocompatibility in various studies, it is important to note that their toxicity might escalate due to successive functionalization. Consequently, a prudent recommendation is to prioritize the utilization of biomimetic molecules, particularly those that have received FDA approval, for any forthcoming therapeutic strategies involving GBNMs. This emphasis is even more significant when GBNMs are modified for multimodal detection and treatment or for addressing cancer through combinatorial techniques [112].

In the upcoming years, it will be crucial to conduct additional research that involves the utilization of FDA-approved modified rGO, the application of NIR-II light sources for irradiation, the incorporation of 3D tumor models for in vitro assays, and the assessment of in vivo toxicity. This collective effort will play a pivotal role in advancing the transition from laboratory testing to eventual clinical applications.

## 5. Conclusions

Graphene oxide and reduced graphene oxide function as unique photothermal agents due to their remarkable structural features. While the materials exhibit comparable structures, their photothermal capacities are very different, affecting the success of photothermal therapy for cancer treatment.

For GO, high concentrations and laser power intensities were necessary to generate the photothermal effect. However, in most cases, the temperature did not surpass 50 °C. Instead, the development of hybrids involving GO and other photothermal materials and anticancer drugs displayed better photothermal effects with similar therapeutic results to those obtained for rGO.

Compared with GO, rGO achieved greater photothermal conversion under the same NIR irradiation conditions. Moreover, a significant decrease in the cell viability of different cancer cell lines treated was found when rGO was used as the PTA. Notably, the chemical reduction method is frequently employed for manufacturing rGO with photothermal characteristics. However, it remains uncertain whether alternative reduction methods could lead to even more substantial NIR absorption and a heightened photothermal effect.

All the GO and rGO-based systems reviewed demonstrated that the heat generated was concentration- and laser-power-intensity dependent. In addition, the use of combined PTT with a secondary therapeutic strategy showed improved therapeutic efficacy compared with that of PTT alone.

Nevertheless, most of the studies reviewed focused on the use of laser light from the NIR-I window, although the NIR-II window offers major benefits, such as a higher penetration depth and the possibility of operating at higher power intensities for irradiation. This aspect should be considered when designing and developing new photothermal strategies and looking for their potential translation to clinical use.

On the other hand, MNPs could be utilized as a guide to a specific location as well as in the photothermal response. Through MNPs, GBNMs could be modified to load drug molecules, making them suitable for drug delivery. The use of MNPs will continue to benefit GBNM for synergistic photothermal therapy.

We hope this review can provide new insights for the future design of GBNM nanoplatforms, especially as photothermal agents in the photothermal treatment of cancer.

## Figures and Tables

**Table 1:** Overview of GO/rGO Hybrids and their Application in Photothermal Cancer Therapy.

GBNM Hybrid System	Cell Line/Cancer Type	Irradiation Conditions	Temperature (°C)	Reference
rGO@AuNS- DODAB/DOPE-FA	Pancreatic	808 nm; 0.1 W/cm^2^	52.6	[79]
(PNIPAMAAM)/GO	hASCs and MDA-MB-231	808 nm; 4.0 W/cm^2^	36	[80]
IR780-NGO-RSV	Ovarian	808 nm; 0.3 W/cm^2^	61.5	[81]
Pd@PPy/GO	MCF-7	808 nm; 1.5 W/cm^2^	30 (ΔT)	[82]
PTX@GO-PEG-OSA	Gastric cancer	808 nm; 1.0 W/ cm^2^	43	[83]
GO–FA/Ce6	MCF-7	808 nm; 2.0 W/cm^2^	17 (ΔT)	[75]
nGO-PEG-ARS	HepG2	808 nm; 2.0 W/cm^2^	60	[73]
fGO@GNRs-DOX	HeLa and A549	808 nm; 1.0 W/cm^2^	59	[71]
GO-PEG-FA	Breast cancer	808 nm; 4.0 W/cm^2^	68	[76]
GO-PEI-GQDs	MDA-MB-231	808 nm; 0.5 W/cm^2^	48	[72]
AGD	A549	808 nm; -	50	[74]
GO+PEGFA+ICG	Ehrlich tumor	808 nm; 1.8 W/cm^2^	40 (ΔT)	[84]
ICG@MS-rGO-FA	Colorectal	808 nm; 1.0 W/cm^2^	26 (ΔT)	[85]
nGO-PEG	Colon	808 nm; 1.5 W/cm^2^	47 (ΔT)	[86]
GO@SiO2@AuNS	KM12C	808 nm; 0.3 W/cm^2^	16 (ΔT)	[87]
nGO-COS-CD47/DTIC	Melanoma	808 nm; 2.0 W/cm^2^	55	[88]
GO-v50-DOX	Melanoma	808 nm; 7.0 W/cm^2^	54	[89]
rGO-AuNS, rGO-AuNR	HUVECs	808 nm; 3.0 W/cm^2^	57 (ΔT)	[90]

Abbreviations: AuNS (gold nano star particle); DODAB/DOPE (bromide/1,2-dioleoyl-sn-glycero-3-phosphoethanolamine); RSV (resveratrol); PNIPAM (poly(N-isopropylacrylamide); AAM (allylamine); PPy (polypyrrole); PTX (paclitaxel); PEG (polyethylene glycol); OSA (oxidized sodium alginate); FA (folic acid); Ce6 (chlorin e6); ARS (artesunate); GNRs (gold nanorods); AGD (graphene oxide (GO)-hybridized nanogels); ICG (indocyanine green); SN (7-ethyl-10-hydroxycamptothecin (SN-38)); MS (mesoporous silica); CD47 (is an antibody); COS (chitosan oligosaccharide); DTIC (dacarbazine); v-50 (azo initiator); AuNR (gold nanorod).

## Data Availability

Not applicable.
